# Ohio Veterinary College

**Published:** 1893-12

**Authors:** 


OHIO VETERINARY COLLEGE.

*(April 5th.—Degree D. V. S. (Doctor Veterinary Science).*

| | |
|---|---|
| Bethune, J. G. | No. Pine Grove, Pa. |
| Boehme, Herman | Newport, Ky. |
| Cantelow, S. H. | Brantford, Ont. |
| Curtis, H. L. | Little Hocking, Ohio. |
| Dixon, C. Price | Cumberland Gap, Tenn. |
| Everly, O. W. | Holmesville, Ohio. |
| Freshour, J. W. | Covington, Ohio. |
| Galbraith, G. R. | Manhattan, Ill. |
| Geary, C. K. | St. Thomas, Ont. |
| Haffman, L. R. | Centreville, Ky. |
| James, T. H. | Maysville, Ky. |
| Marshall, H. | Cumberland Gap, Tenn. |
| Miller, Alvin | Chillicothe, Ohio. |
| McCann, A. A. | River Dissert, Quebec. |
| Nesbitt, J. G. | Ottawa, Ont. |
| Ruth, C. V. | Torch, Ohio. |
| Smiser, H. A. | Cynthiana, Ky. |
| Spitler, J. L. | Dayton, Ohio. |
| Ware, J. T. | Paris, Ky. |
| Willerton, Tom | Lynville, Ill. |

